# Network Pharmacology-Based Strategy for Predicting Therapy Targets of Traditional Chinese Medicine Xihuang Pill on Liver Cancer

**DOI:** 10.1155/2020/6076572

**Published:** 2020-03-14

**Authors:** Xu Zhao, Jian Hao, Sinuan Chen

**Affiliations:** ^1^Department of Traditional Chinese Medicine, Shenzhen Longgang Central Hospital, Shenzhen, Guangdong Province 518116, China; ^2^Clinical Cancer Therapy Center, The Fourth Central Hospital of NanKai University, Hebei District, 300140 Tianjin, China

## Abstract

**Objective:**

To investigate the potential therapy targets and pharmacological mechanism of traditional Chinese medicine (TCM) Xihuang pill in liver cancer based on network pharmacology.

**Methods:**

Drug ingredients-target network was constructed based on the target sets of Xihuang pill and liver cancer. The overlapping genes between Xihuang pill targets and liver cancer-related molecular targets were investigated using comparative analysis. Moreover, the PPI network and module was constructed based on overlapping genes and hub nodes, respectively, followed by the pathway enrichment analysis.

**Results:**

A drug ingredients-target network was established with 1184 nodes and 11035 interactions. Moreover, a total of 106 overlapping genes were revealed between drug targets and liver cancer molecular targets. Furthermore, a PPI network and 4 modules were further investigated based on overlapping genes, respectively. These hub nodes such as VEGFA and EGFR were mainly enriched in GO functions including positive regulation of MAP kinase activity, activation of protein kinase activity, regulation of MAP kinase activity, and pathways like proteoglycans in cancer, bladder cancer, and estrogen signaling.

**Conclusion:**

VEGFA and EGFR might be potential therapy targets of Xihuang pill in liver cancer. Furthermore, the effect of Xihuang pill on liver cancer might be realized by targeting VEGFA and EGFR in pathways like proteoglycans in cancer and estrogen signaling.

## 1. Introduction

As the second leading cause of death, liver cancer has caused a wide social burden for a long period of time [1]. Although partial surgical resection is the optimal therapy strategy for patients with liver cancer, the recurrence rates after surgery are still very high [2, 3]. Thus, exploring the effective clinical treatment for liver cancer is necessary.

Traditional Chinese medicine (TCM) has been widely used for clinical treatment of various tumors such as liver cancer [4, 5]. Xihuang pill is a complementary and alternative medicine that has been used in TCM due to the inhibition for tumor cell proliferation [6]. Xihuang pill is composed of Ru Xiang (olibanum), Mo Yao (*Commiphora myrrha*), She Xiang (Moschus), and Niu Huang (calculus bovis), which exert multiple antitumor effects [7]. A previous study shows that Xihuang pill has an anticancer effect on breast tumor [8], gastric cancer, and primary liver cancer [7]. Actually, Xihuang pill could improve quality of life and clinical manifestations of advanced primary liver cancer patients [9]. A previous study indicates that there is a reversal effect of serum containing Xihuang pill on the multidrug resistance of liver cancer cells via P-glytoprotein pathway [10]. Although Xihuang pill is effective in treatment of liver cancer, the drug targets and pharmacological mechanisms of Xihuang pill on liver cancer are still unclear.

The network pharmacology analysis is a useful tool for further understanding of the drug action [11], which may provide insights into how we can improve drug discovery for complex diseases [12]. In the current study, the potential mechanism of Xihuang pill on the treatment of liver cancer was analyzed by a network-based systematic study (as shown in Supplementary [Supplementary-material supplementary-material-1]). Briefly, the drug ingredients-target network was constricted based on the target sets of Xihuang pill and liver cancer. Then, the overlapping genes were investigated using comparative analysis. Moreover, the networks of protein-protein interaction (PPI) and modules were constructed based on overlapping genes and hub nodes, followed by the GO and KEGG pathway enrichment analysis. We hoped to explore the potential therapy targets of Xihuang pill in the treatment of liver cancer, which can provide the basis for the pharmacological mechanism study of Xihuang pill.

## 2. Materials and Methods

### 2.1. Identification of Ingredients of Drug

The chemical ingredients of Xihuang pill were collected from the Traditional Chinese Medicine Systems Pharmacology (TCMSP) Database (http://lsp.nwsuaf.edu.cn/tcmsp.php) [13] and the Traditional Chinese Medicine Integrated Database (TCMID, http://www.megabionet.org/tcmid/) [14]. According to the relevant parameters of the pharmacokinetic properties, the ingredients were screened according to oral bioavailability (OB) and drug-likeness (DL) values and those ingredients with DL ≥ 0.18 and OB ≥ 30% were selected as the active ingredients [15, 16].

### 2.2. Drug Target

Usually, the ingredients of drugs play a role in related biological functions via targets. To predict the targets of ingredients of Xihuang pill, the small molecular structure information of active ingredients in Xihuang pill was retrieved on the PubChem database (https://pubchem.ncbi.nlm.nih.gov/) [17]. Subsequently, the targets were screened with a screening online tool called the Swiss Target Prediction (http://www.swisstargetprediction.ch/) [18]. Finally, all the target information was standardized using UniProt (http://www.UniProt.org/).

### 2.3. Drug Ingredients-Target Network Construction

The drug ingredients-target network was constructed by Cytoscape software (version: 3.0.0, http://chianti.ucsd.edu/cytoscape-3.0.0/) [11]. The topological parameters of the network, including the degree were analyzed using Degree Centrality (DC) based on CytoNCA software [19].

### 2.4. Liver Cancer Targets

The main source of disease targets for liver cancer was obtained from IPA (http://www.ingenuity.com). The targets of liver cancer were retrieved after deleting duplicate data. Then, the target datasets were compared by Geneweaver (http://www.geneweaver.org) [20], and the overlapping targets of both liver cancer and the drug ingredients were considered potential targets of Xihuang pill for the treatment of liver cancer.

### 2.5. PPI Network Construction and Module Analysis

According to STING database (http://www.string-db.org/) [21] with the species limited to “*Homo sapiens*” and the confidence score >0.9, the protein interaction pairs associated with overlapping target genes between Xihuang pill and liver cancer were screened. And the PPI network was constructed via utilizing the network visualization software Cytoscape (version: 3.0.0, http://chianti.ucsd.edu/cytoscape-3.0.0/) [11], and the topological properties of the PPI network were analyzed through NetworkAnalyzer (default settings). Furthermore, MCODE (version 1.5.1) [22] was used to screen the significant enriched modules from the PPI network.

### 2.6. Hub Gene Investigation in PPI Network

The hub genes in PPI network were further investigated by a Cytoscape plugin cytoHubba [23], and 11 network topology parameters, including Degree, Edge Percolated Ingredients, Maximum Neighborhood Ingredients, Density of Maximum Neighborhood Ingredients, Maximal Clique Centrality Bottleneck, EcCentricity, Closeness, Radiality, Betweenness, and Stress were selected for importance calculation. After screening the core nodes in PPI network, the rank sum ratio (RSR) [24] was used to rank the nodes synthetically to get the core nodes of the network. The more important the output of a node, the higher the value of this node in the network.

### 2.7. Enrichment Analysis for the Hub Genes

In order to reveal the potential biological function of Xihuang pill in the treatment of liver cancer, GO (Gene Ontology) functions [25] and KEGG (Kyoto Encyclopedia of Genes and Genomes) pathway [26] enrichment analysis were performed. Specially, GO terms were grouped into the following three categories: biological process (BP), molecular function (MF), and cellular component (CC) [25]. And the KEGG analysis was conducted according to the enrichment analysis tool of the Database for Annotation, Visualization and Intergrated Discovery (DAVID, version: 6.8) software [27]. A *p* value <0.05 was considered as the cutoff criterion.

## 3. Results

### 3.1. Drug Ingredients-Target Network Analysis

**Figure 1:**
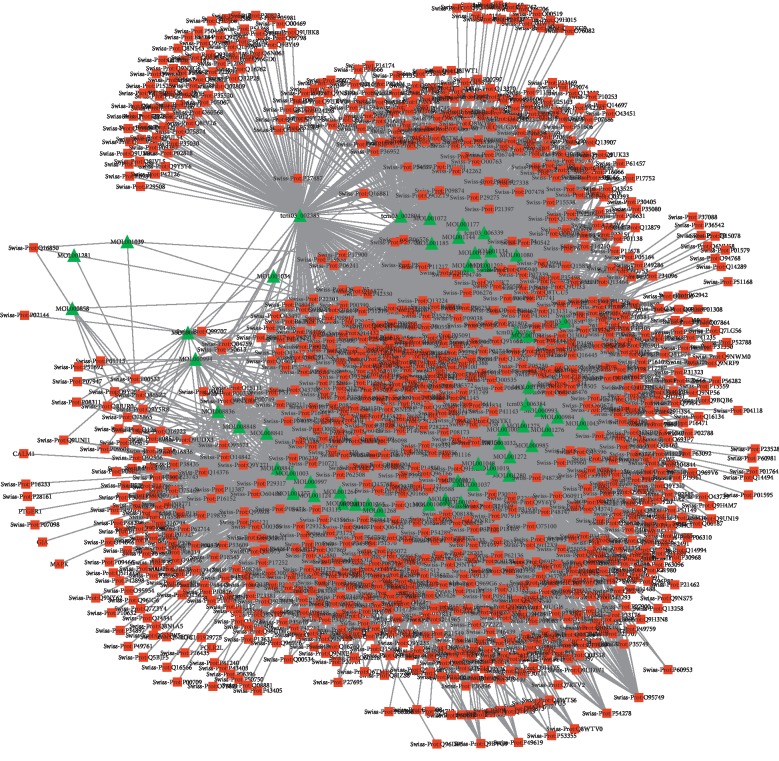
The drug ingredients-target network in the current study. The red square represented the target; the green triangle represented the drug ingredient; the line between two nodes represented the interaction.

**Figure 2:**
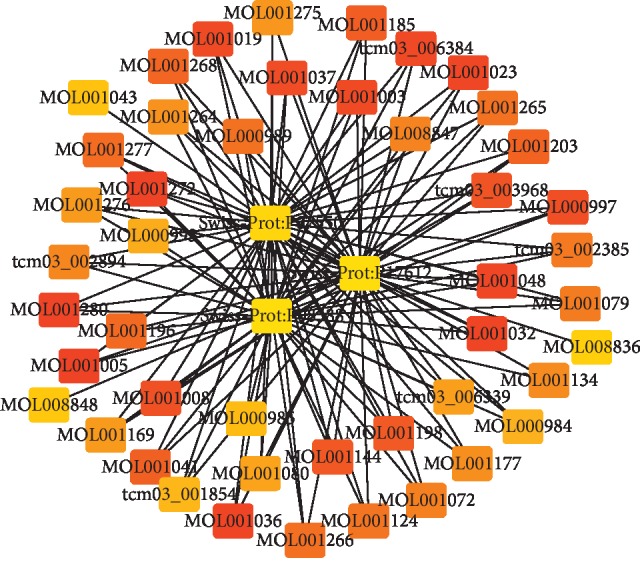
The module constructed by the TOP 50 nodes from the drug ingredients-target network. The triangle represented the ingredients of drug, and the square represented the targets of disease. The darker the color, the more significant it is.

A total of 53 ingredients and 1131 targets of Xihuang pill, as well as 566 molecular targets of liver cancer, were obtained in the current study. Based on these data, the drug ingredients-target network was constructed. As shown in Figure 1, the network consists of 1184 nodes and 11035 interactions (see details in Supplementary [Supplementary-material supplementary-material-1]). Moreover, the top 50 nodes including 3 targets were further selected to construct the module of the drug ingredients-target network (Figure 2; Supplementary [Supplementary-material supplementary-material-1]).

### 3.2. Overlapping Genes between Drug Targets and Liver Cancer Molecular Targets

After the comparative analysis, the overlapping genes, which might be potential drug therapy target for Xihuang pill in liver cancer, between drug targets and liver cancer therapy targets were obtained. The result showed that a total of 106 overlapping genes (Attachment 1), including VEGFA, EGFR, ESR1, PLG, and MAPK3, were revealed in the current study.

Additionally, the KEGG pathway enrichment analysis showed that these overlapping genes were mainly enriched in pathways, such as metabolic pathways, pathways in cancer, proteoglycans in cancer, estrogen-signaling pathway, and HIF-1-signaling pathway. The top 20 pathways enriched by overlapping genes were listed in Table 1.

### 3.3. PPI Network Analysis

**Figure 3:**
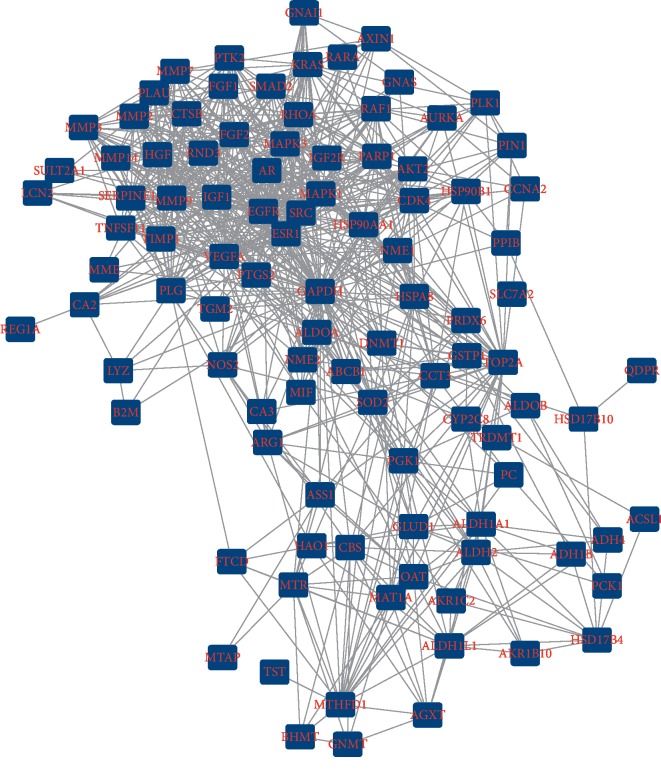
The protein-protein interaction network. The PPI network constructed by the potential therapy targets of Xihuang pill on liver cancer; the square represented the targets; the line between two nodes represented the interaction.

**Figure 4:**
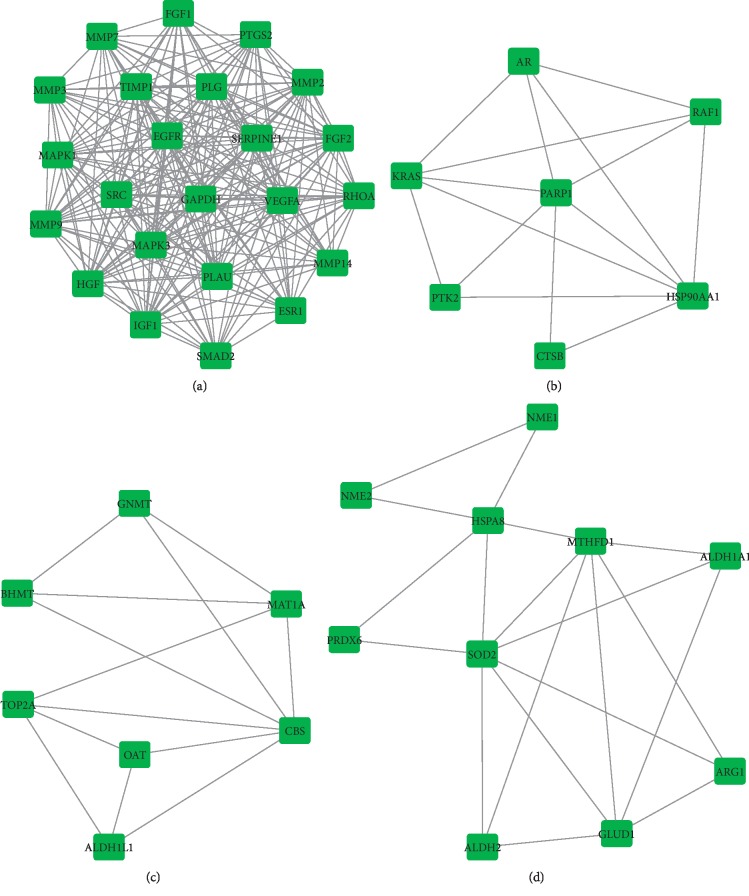
The modules networks of PPI. The square represented the targets; the line between two nodes represented the interaction. (a) Module 1. (b) Module 2. (c) Module 3. (d) Module 4.

Based on the potential pharmacodynamic target of Xihuang pill for liver cancer, a PPI network was constructed by using the STRING. The result showed that there were 102 nodes and 766 interactions in the network (Figure 3). Moreover, a total of 4 modules including module 1 (score = 9.913), module 2 (score = 2.143), module 3 (score = 1.5), and module 4 (score = 1.333) were further investigated from PPI network using MOCDE (Figures 4(a)–4(d)).

### 3.4. Hub Nodes Investigation

**Figure 5:**
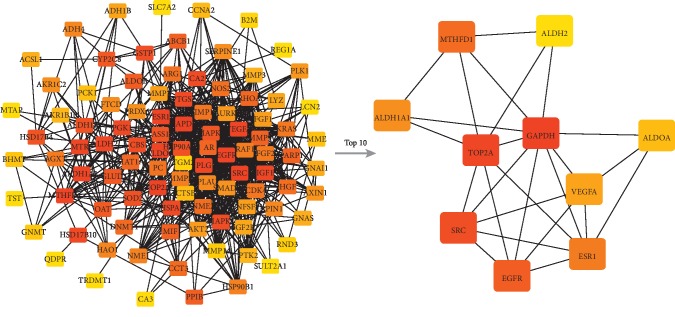
The results of hub nodes in current PPI network. The ranking of hub nodes based on median. From red to yellow represented a decline in importance; the line between two nodes represented the interaction.

**Figure 6:**
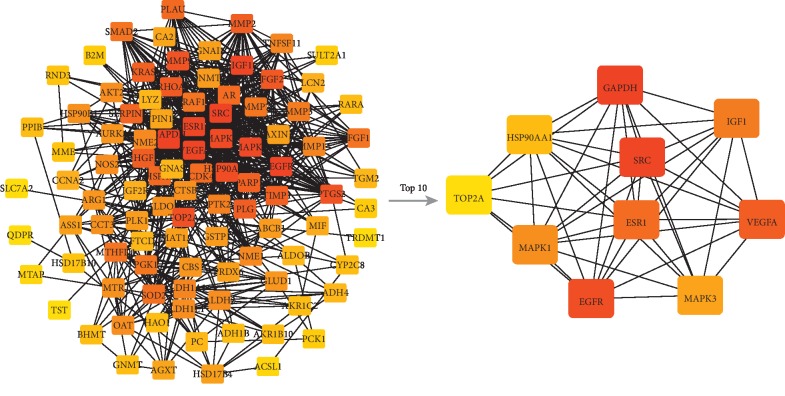
The results of hub nodes in current PPI network. The ranking of hub nodes based on degree. From red to yellow represented a decline in importance; the line between two nodes represented the interaction.

According to the PPI network, the core nodes of the network were screened based on the characteristics of the network's topological structure (Figures 5 and 6). The top 20 important nodes including VEGFA (RSR = 0.1218), EGFR (RSR = 0.1382), ESR1 (RSR = 0.1427), PLG (RSR = 0.1436), and MAPK3 (RSR = 0.1464) were summarized in Table 2. Moreover, the association among top 5 hub nodes, drug ingredients, and CTM was listed in Table 3.

### 3.5. Investigation of Pathways Enriched by Hub Nodes

**Figure 7:**
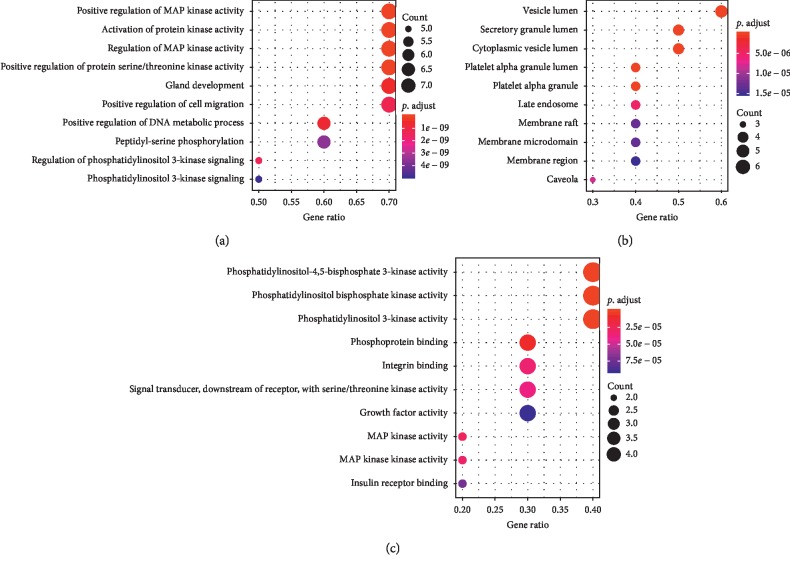
Gene ontology (GO) term enrichment for hub genes. GO terms including biological process (BP), cellular component (CC), and molecular function (MF) enriched by hub genes, respectively.

The top 5 hub nodes including VEGFA, EGFR, ESR1, PLG, and MAKP3 were used for further GO function and pathway enrichment analyses. The result showed that the hub genes could be assigned to different GO terms for BP, CC, and MF categories. The prominent functions enriched by hub genes were positive regulation of MAP kinase activity, activation of protein kinase activity, regulation of MAP kinase activity and phosphatidylinositol bisphosphate kinase activity, of which the top 10 functions for GO terms are presented in Figure 7. Moreover, these hub nodes were mainly enriched in pathways like proteoglycans in cancer (ID: 05205; count = 4; FDR = 8.93E-06), bladder cancer (ID: 05219; count = 3; FDR = 8.93E-06), and estrogen-signaling pathway (ID: 04915; count = 3; FDR = 6.48E-05). The top 10 pathways are listed in Table 4.

## 4. Discussion

Liver cancer is one of the most lethal cancers having worldwide prevalence, and the effect of clinical treatment for this disease is not satisfactory [28]. The current study, for the first time, explored the potential therapy targets of Xihuang pill on liver cancer based on network pharmacology analysis. The results showed that a drug ingredients-target network was established with 1184 nodes and 11035 interactions. Moreover, a total of 106 overlapping genes were revealed between drug targets and liver cancer molecular targets. Furthermore, a PPI network and 4 modules networks were further investigated based on overlapping genes and hub nodes, respectively. These hub nodes such as VEGFA, EGFR, ESR1, PLG, and MAPK3 were mainly enriched in pathways like proteoglycans in cancer, bladder cancer, and estrogen-signaling pathway.

Vascular endothelial growth factor (VEGF) family plays a major role in angiogenesis, which are essential for both healing of injured tissue and proliferation of carcinoma cells [29]. The previous meta-analysis indicates that there is a prognostic role for VEFGA in various cancers including cervical cancer, gastric cancer, epithelial ovarian cancer, and liver cancer [30–33]. Taniguchi et al. indicates that VEGF promotes proliferation of hepatocytes through reconstruction of liver sinusoids by proliferation of sinusoidal endothelial cells [33]. Importantly, VEGF is an important drug target of various TCM [34, 35]. A previous study shows that the TCM Fuzhenghuayu has a decoction effect on VEGF secretion in hepatic stellate cells [36]. However, the association between VEGF and Xihuang pill in liver cancer is unknown. Moreover, epidermal growth factor receptor (EGFR) is a transmembrane protein associated with the epidermal growth factor family [37]. The compartmentalization and biological function of EGFR in liver plasma membrane have been focused [38]. A previous study indicates that there is an increment of serum EGFR level in liver cancer patients [39]. Actually, there is a dual role of EGFR in liver injury and regeneration after acetaminophen overdose in mice [40]. Zhu et al. indicate that the EGFR status is independently correlated with TCM treatment in non-small-cell lung cancer patients [41]. Although previous study has confirmed the relation between EGFR expression and TCM active compounds using cell membrane chromatography [42], the effect of Xihuang pill on EGFR expression in liver cancer is still unclear. In the current study, VEGFA and EGFR were not only overlapping genes revealed between Xihuang pill target sets and liver cancer target sets but also hub nodes in PPI modules. Thus, we speculated that genes such as VEGFA and EGFR might be potential therapy targets of Xihuang pill in liver cancer.

Proteoglycans perform multiple functions in cancer and angiogenesis due to their polyhedric nature [43]. The deterioration of liver function is accompanied by an increase in the amount of chondroitin sulfate proteoglycans [44]. The alteration of proteoglycan composition interferes with the physiologic function of the liver on several levels [45]. Actually, the biological function of proteoglycans has been widely investigated in VEGF-induced diseases [46, 47]. A previous study shows that the activation of EGFR contributes to the inhibition of axon regeneration [48]. Furthermore, estrogen plays a vital role in the progression of liver cancer [49]. Kim et al. indicate that estrogen-related receptor γ is upregulated in liver cancer [50]. Previous studies show that VEGF and EGFR expression are all associated with estrogen receptor status in patients with cancer [51, 52]. Meanwhile, cytoplasmic expression of estrogen receptor can predict poor outcome of EGFR-TKI therapy in metastatic lung adenocarcinoma [53]. In this study, the pathway analysis showed that the potential targets such as VEGFA and EGFR were mainly enriched in pathways including proteoglycans in cancer and estrogen-signaling pathway. Thus, we speculated that the effect of Xihuang pill on liver cancer might be realized by targeting VEGFA and EGFR in pathways like proteoglycans in cancer and estrogen signaling. However, there were some limitations in this study such as small sample size and lack of verification analysis. Thus, a further verification study based on a large sample size is needed.

In conclusion, VEGFA and EGFR might be potential therapy targets of Xihuang pill in liver cancer. Furthermore, the effect of Xihuang pill on liver cancer might be realized by targeting VEGFA and EGFR in pathways like proteoglycans in cancer and estrogen signaling.

## Figures and Tables

**Table 1:** The top 20 pathways enriched by the overlapping genes between Xihuang pill targets and liver cancer molecular targets.

ID	Pathway description	Count	FDR
1100	Metabolic pathways	35	2.81*E* − 16
5200	Pathways in cancer	24	4.18*E* − 19
5205	Proteoglycans in cancer	18	4.38*E* − 15
4151	PI3K-Akt-signaling pathway	15	4.16*E* − 09
4915	Estrogen-signaling pathway	13	9.30*E* − 14
4015	Rap1-signaling pathway	13	1.04*E* − 09
4066	HIF-1-signaling pathway	12	9.29*E* − 12
4510	Focal adhesion	11	1.12*E* − 07
4014	Ras-signaling pathway	11	2.20*E* − 07
1230	Biosynthesis of amino acids	10	9.36*E* − 11
5218	Melanoma	10	9.36*E* − 11
5215	Prostate cancer	10	5.39*E* − 10
4068	FoxO-signaling pathway	10	1.29*E* − 08
5206	MicroRNAs in cancer	10	5.79*E* − 08
5219	Bladder cancer	8	4.16*E* − 10
4370	VEGF-signaling pathway	8	1.34*E* − 08
5212	Pancreatic cancer	8	1.64*E* − 08
4914	Progesterone-mediated oocyte maturation	8	1.12*E* − 07
4912	GnRH-signaling pathway	8	2.20*E* − 07
5210	Colorectal cancer	7	2.20*E* − 07

Count, the number of genes enriched in certain pathways; FDR, false discovery rate.

**Table 2:** The top 20 core targets genes in the drug-target gene network.

Name	RSR
VEGFA	1.22*E* − 01
EGFR	1.38*E* − 01
ESR1	1.43*E* − 01
PLG	1.44*E* − 01
MAPK3	1.46*E* − 01
IGF1	1.49*E* − 01
GAPDH	1.54*E* − 01
SRC	1.55*E* − 01
MAPK1	1.84*E* − 01
HGF	1.88*E* − 01
PTGS2	1.90*E* − 01
MMP9	2.08*E* − 01
FGF2	2.17*E* − 01
TIMP1	2.25*E* − 01
MMP2	2.25*E* − 01
SERPINE1	2.33*E* − 01
HSP90AA1	2.35*E* − 01
TOP2A	2.44*E* − 01
RHOA	2.47*E* − 01

RSR, the rank sum ratio.

**Table 3:** The drug ingredients corresponding to hub nodes.

Hub nodes	Code	CTM	Ingredients
VEGFA	Swiss-Prot:P15692	Niuhuang	MOL008847
VEGFA	Swiss-Prot:P15692	Ruxiang	MOL001280
VEGFA	Swiss-Prot:P15692	Ruxiang	MOL001264
VEGFA	Swiss-Prot:P15692	Ruxiang	MOL001266
VEGFA	Swiss-Prot:P15692	Ruxiang	MOL001265
VEGFA	Swiss-Prot:P15692	Ruxiang	MOL001268
VEGFA	Swiss-Prot:P15692	Ruxiang	MOL001277
VEGFA	Swiss-Prot:P15692	Moyao	MOL001003
VEGFA	Swiss-Prot:P15692	Moyao	MOL000997
VEGFA	Swiss-Prot:P15692	Moyao	MOL001023
VEGFA	Swiss-Prot:P15692	Moyao	MOL001005
VEGFA	Swiss-Prot:P15692	Moyao	MOL000989
VEGFA	Swiss-Prot:P15692	Moyao	MOL001124
EGFR	Swiss-Prot:P00533	Niuhuang	MOL008847
EGFR	Swiss-Prot:P00533	Ruxiang	MOL000858
EGFR	Swiss-Prot:P00533	Ruxiang	MOL001281
EGFR	Swiss-Prot:P00533	Moyao	MOL001003
EGFR	Swiss-Prot:P00533	Moyao	MOL000989
EGFR	Swiss-Prot:P00533	Moyao	MOL001039
ESR1	Swiss-Prot:P03372	Shexiang	tcm03_006384
ESR1	Swiss-Prot:P03372	Shexiang	tcm03_006339
ESR1	Swiss-Prot:P03372	Shexiang	tcm03_001854
ESR1	Swiss-Prot:P03372	Shexiang	tcm03_003968
ESR1	Swiss-Prot:P03372	Niuhuang	MOL008836
ESR1	Swiss-Prot:P03372	Ruxiang	MOL001276
ESR1	Swiss-Prot:P03372	Ruxiang	MOL001272
ESR1	Swiss-Prot:P03372	Ruxiang	MOL001275
ESR1	Swiss-Prot:P03372	Ruxiang	MOL001264
ESR1	Swiss-Prot:P03372	Ruxiang	MOL001266
ESR1	Swiss-Prot:P03372	Ruxiang	MOL001265
ESR1	Swiss-Prot:P03372	Ruxiang	MOL001268
ESR1	Swiss-Prot:P03372	Ruxiang	MOL001277
ESR1	Swiss-Prot:P03372	Moyao	MOL001203
ESR1	Swiss-Prot:P03372	Moyao	MOL001198
ESR1	Swiss-Prot:P03372	Moyao	MOL001003
ESR1	Swiss-Prot:P03372	Moyao	MOL000997
ESR1	Swiss-Prot:P03372	Moyao	MOL001032
ESR1	Swiss-Prot:P03372	Moyao	MOL001036
ESR1	Swiss-Prot:P03372	Moyao	MOL001048
ESR1	Swiss-Prot:P03372	Moyao	MOL001023
ESR1	Swiss-Prot:P03372	Moyao	MOL001037
ESR1	Swiss-Prot:P03372	Moyao	MOL001019
ESR1	Swiss-Prot:P03372	Moyao	MOL001005
ESR1	Swiss-Prot:P03372	Moyao	MOL001008
ESR1	Swiss-Prot:P03372	Moyao	MOL001041
ESR1	Swiss-Prot:P03372	Moyao	MOL001196
ESR1	Swiss-Prot:P03372	Moyao	MOL000989
ESR1	Swiss-Prot:P03372	Moyao	MOL001079
ESR1	Swiss-Prot:P03372	Moyao	MOL001124
ESR1	Swiss-Prot:P03372	Moyao	MOL001134
ESR1	Swiss-Prot:P03372	Moyao	MOL001072
ESR1	Swiss-Prot:P03372	Moyao	MOL001080
ESR1	Swiss-Prot:P03372	Moyao	MOL000993
PLG	Swiss-Prot:P00747	Shexiang	tcm03_002894
PLG	Swiss-Prot:P00747	Shexiang	tcm03_002385
MAPK3	Swiss-Prot:P27361	Ruxiang	MOL001266
MAPK3	Swiss-Prot:P27361	Ruxiang	MOL001265
MAPK3	Swiss-Prot:P27361	Ruxiang	MOL001268
MAPK3	Swiss-Prot:P27361	Moyao	MOL000997
MAPK3	Swiss-Prot:P27361	Moyao	MOL001036
MAPK3	Swiss-Prot:P27361	Moyao	MOL001023
MAPK3	Swiss-Prot:P27361	Moyao	MOL001037
MAPK3	Swiss-Prot:P27361	Moyao	MOL001019
MAPK3	Swiss-Prot:P27361	Moyao	MOL001124

CTM, Chinese traditional medicine; VEGFA, vascular endothelial growth factor A; EGFR, epidermal growth factor receptor; ESR1, estrogen receptor 1; PLG, plasminogen; MAPK3, mitogen-activated protein kinase 3.

**Table 4:** The top 10 pathways enriched by the top 5 hub nodes in PPI network.

ID	Description	Count	FDR
05205	Proteoglycans in cancer	4	8.93*E* − 06
05219	Bladder cancer	3	8.93*E* − 06
05212	Pancreatic cancer	3	2.40*E* − 05
04915	Estrogen-signaling pathway	3	6.48*E* − 05
04066	HIF-1-signaling pathway	3	7.06*E* − 05
04014	Ras-signaling pathway	3	3.80*E* − 04
04015	Rap 1-signaling pathway	3	3.80*E* − 04
04320	Dorso-ventral axis formation	2	3.80*E* − 04
04510	Focal adhesion	3	3.80*E* − 04

FDR, false discovery rate; Count, the number of nodes enriched in certain pathway.

## Data Availability

The data used to support the findings of this study are available from the corresponding author upon request.

## Additional Points

*Highlights*. (1) VEGFA and EGFR might be potential therapy targets of Xihuang pill in liver cancer. (2) Proteoglycans in cancer and estrogen signaling might be associated with liver cancer. (3) Totally, 106 genes were explored between drug targets and liver cancer targets

## Supplementary Materials

Supplementary Materials Supplementary Figure 1: workflow of network pharmacology analysis. Supplementary File 1: all nodes and interactions in drug ingredients-target network. Supplementary File 2: the top 50 nodes in drug ingredients-target network.
