# Supplementary figures and images for: Network Pharmacology-Based Strategy for Predicting Therapy Targets of Traditional Chinese Medicine Xihuang Pill on Liver Cancer
Source: Evid Based Complement Alternat Med. 2020 Mar 14;2020:6076572. doi: 10.1155/2020/6076572 (PMC7102465; doi:10.1155/2020/6076572)

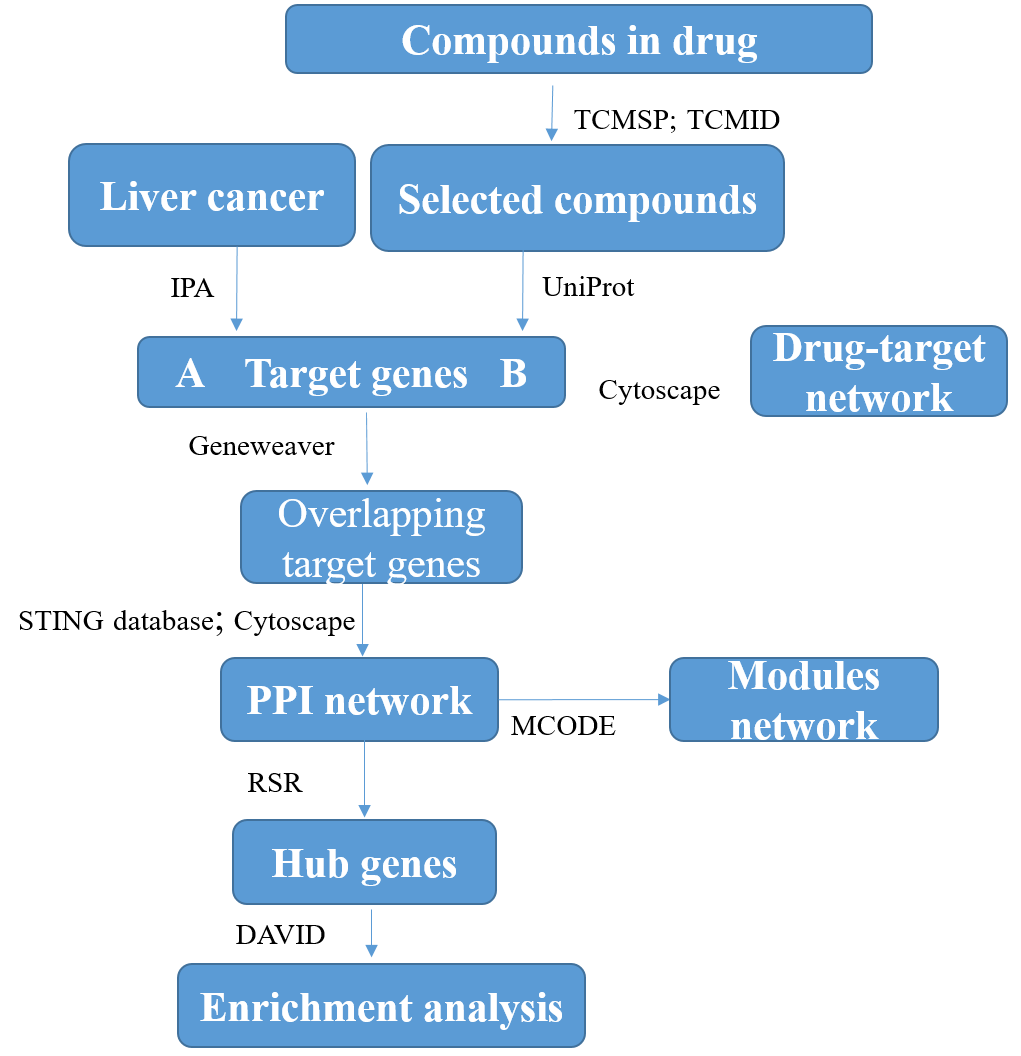

Supplement: Supplementary Materials — Supplementary Figure 1: workflow of network pharmacology analysis. Supplementary File 1: all nodes and interactions in drug ingredients-target network. Supplementary File 2: the top 50 nodes in drug ingredients-target network. [file 6076572.f1.zip › 6076572.f1/Figure and Supplementary files/Supplementary figure 1-revised.tif]
